# Diagnostic Investigation of Two Cases of *Oslerus osleri* Infection in Dogs: Microscopic Challenges and Molecular Characterisation

**DOI:** 10.1002/vms3.70970

**Published:** 2026-04-29

**Authors:** Jeremie Korchia, Vishnu Manikantan, Ashley K. McGrew, Christina M. Jeffries, Rebecca L. Makii, Kelly McCord, Sriveny Dangoudoubiyam

**Affiliations:** ^1^ Department of Pathobiology and Diagnostic Investigation, College of Veterinary Medicine Michigan State University East Lansing Michigan USA; ^2^ Department of Comparative Pathobiology, College of Veterinary Medicine Purdue University West Lafayette Indiana USA; ^3^ Department of Microbiology, Immunology and Pathology, College of Veterinary Medicine & Biomedical Sciences Colorado State University Fort Collins Colorado USA; ^4^ Royal Vista Veterinary Specialists Windsor Colorado USA

**Keywords:** Baermann technique, bronchoalveolar lavage cytology, canine, lungworm, PCR, verminous pneumonia

## Abstract

**Background:**

The diagnosis of certain lungworm infections in dogs relies heavily on bronchoalveolar lavage (BAL) cytology or lesion histopathology, especially in situations in which faecal diagnostics and bloodwork may have limited value. Differentiating morphologically similar parasitic larvae on BAL cytology, such as *Oslerus osleri* or *Filaroides hirthi*, may be challenging, and molecular approaches are not yet widely available.

**Objective:**

Description of two cases of *O. osleri* infection in young dogs, including (1) representative examples of BAL cytology and lesion‐specific histopathologic findings and (2) molecular characterisation, based on targeted gene amplification and sequencing, from cytologic and histopathologic samples of *O. osleri*.

**Methods:**

Two cases of *O. osleri* infection were investigated and followed in detail. BAL slides and tracheobronchial biopsy samples were analysed: six primer pairs spanning the ribosomal RNA gene of *O. osleri* were used to amplify the specific regions of genomic DNA from stained BAL slides (Case 1) and from formalin‐fixed paraffin‐embedded (FFPE) tracheobronchial biopsy (Case 2). Amplicon sequences were compared to *O. osleri* nucleotide sequences available in GenBank.

**Results:**

The amplicon sequences obtained from both cases were 100% identical, indicating that the infections in both dogs were caused by the same organism. BLAST analysis against the GenBank nucleotide database showed a 100% match with *O. osleri*, thereby confirming the diagnosis.

**Conclusions:**

PCR testing for *O. osleri* is feasible on genomic DNA obtained from stained BAL smears and sections of FFPE tissue. Further testing of these primers on other lungworms, such as *F. hirthi*, is warranted.

## Graphical abstract

- Two cases of young dogs with chronic cough were investigated, one with bronchoalveolar lavage for cytology, the other with endoscopic biopsy for histopathology.
- DNA was extracted from stained smears and from formalin paraffin‐embedded blocs, six primer pairs spanning the ribosomal RNA gene of O. osleri were designed and amplicons were sequenced, resulting in 100% homology between the 2 cases and with the available sequences of O. osleri in GenBank.
- of O. osleri infection in young dogs, including 1) representative examples of BAL cytology and lesion‐specific histopathologic findings, and 2) molecular characterization, based on targeted gene amplification and sequencing, from cytologic and histopathologic samples of O. osleri.
- This demonstrates the feasibility of PCR for O. osleri (not commonly commercially available) from typical cytology and histopathology samples, consisting of a significant asset for specification in worm closely resembling F. hirthi, and in a disease for which blood work and fecal testing are usually of limited usefulness.

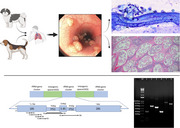

## 1 Introduction

*Oslerus* (*Filaroides*) *osleri* is a ubiquitous nematode affecting domestic and wild canids. Infections by *O. osleri* have been described in numerous canine breeds representing various ages (Yao et al. 2011). However, infection is commonly reported in young dogs less than 2 years of age and is often clinically misdiagnosed as kennel cough (Yao et al. 2011). After becoming infected with *O. osleri*, dogs often show signs of tracheobronchitis accompanied by a prolonged cough and may suffer from respiratory distress (Rafailov et al. 2021). Characteristic nodules typically develop at the bifurcation of the trachea, although there have been retrospective studies where lower respiratory tract disease has been attributed to *O. osleri* infection (Brownlie 1990).

*Oslerus osleri* has a direct life cycle within canids, with the infective first‐stage larvae (L1) released into the environment, primarily through faeces and occasionally via vomitus or saliva. Ingestion of larvae leads to direct, immediate infection of a subsequent host. This contributes, in part, to the relatively easy spread of the infection, compared to life cycles requiring an intermediate host (Conboy and Jane 2021). The intermittent shedding of larvae is acknowledged as a cause for imperfect sensitivity of single‐approach faecal testing (Conboy and Jane 2021).

Morphologically, *O. osleri* adults are described as ranging from 9 to 15 mm in length for females and from 5 to 6 mm for males (Barr and Bowman 2011; King 2004; Morgan 1992; Streeter 2007). The earliest time frame in which a diagnosis is possible is approximately 2.5 months postinfection. A diagnosis may be made by the detection of L1 larvae in the faeces or in bronchial secretions. However, faecal screening may be of low sensitivity for *Filaroides* nematodes broadly speaking. Moreover, subtyping L1 larvae of *Filaroides* nematodes based on morphology alone may prove to be particularly complex, so that a diagnosis of *O. osleri* infection often involves endoscopic detection of the characteristic nematode‐filled submucosal nodules located at the tracheal bifurcation or histologic examination (Yao et al. 2011). Prognoses and treatment recommendations vary depending on sources, but sources converge in recommending nematode species identification.

Given the diagnostic challenges associated with this disease, alternative or additional diagnostic approaches may be warranted to investigate cases with non‐specific clinical presentations and further characterise the involved parasite(s). In this study, we report the cases of two young dogs in which the diagnostic investigation was performed through cytologic evaluation of bronchoalveolar lavage (BAL) fluid and through histopathology, respectively. A PCR assay was designed for *O. osleri* on BAL and FFPE samples. We intend to illustrate the cytologic challenges in identifying *O. osleri* versus other related lungworms (e.g., *Filaroides hirthi*), especially when observing the immature life stages. We also intend to demonstrate that PCR testing for *O. osleri* could be performed on both fresh and fixed specimens (stained dry‐mount smears and FFPE slices), thereby making it a particularly relevant and polyvalent diagnostic tool for use in a clinical context.

## 2 Case 1

### 2.1 Clinical Report

A 2.5‐year‐old spayed female English Bulldog was presented to a veterinary clinic for chronic cough and haemoptysis. Reportedly, she was also exposed to another dog diagnosed with kennel cough. The patient had a history of chronic dermatologic disease attributed to environmental allergy and was managed with weekly baths, immunotherapy injections (Heska) for desensitisation by allergenic extracts and oral immunosuppressive therapy consisting of daily oclacitinib (Apoquel), an immune suppressant drug in the Janus kinase (JAK) inhibitor class (0.2–0.8 mg/kg/day depending on clinical signs) and repeated courses of tapered steroid (prednisone, starting at 0.8 mg/kg/day PO and then tapered). Coughing was especially present in phases of excitement, but whether the persistent cough was related to allergies was unclear. The physical examination revealed a faint, dry, non‐productive cough every few seconds. Radiographs of the upper airway revealed a soft tissue opacity/swelling in the throat/larynx area, with primary differential diagnoses including a laryngeal abscess or neoplasm. The patient was prescribed a course of doxycycline and a tapering dose of prednisone (starting at 1.1 mg/kg/day PO and tapered over 30 days). Follow‐up cervical radiographs obtained 10 days later revealed a marked reduction in lesion size, and the prednisone taper was continued. However, shortly after completion of corticosteroid therapy, clinical signs worsened, prompting referral to an internal medicine specialist.

On admission to internal medicine consultation, harsh lung sounds were detected in all lung fields. Haematology (ProCyte Dx; IDEXX Laboratories) and serum biochemistry (Catalyst Dx; IDEXX Laboratories) panels were unremarkable, apart from mild eosinophilia (1.88 × 10^9^/L, reference interval [RI]: 0.06–1.23 × 10^9^/L). As part of the standard diagnostic workup, a SNAP 4Dx Plus test (IDEXX Laboratories) was performed, and neither antigens for heartworm nor antibodies for tickborne agents were detected. Subsequently, a bronchoscopy and BAL were performed. At the time of the BAL interpretation, bronchoscopy findings were not communicated to the pathologists; retrospectively, the trachea was grossly described as hyperaemic, with numerous nodules containing nematodes and a moderate amount of mucus.

**FIGURE 1:**
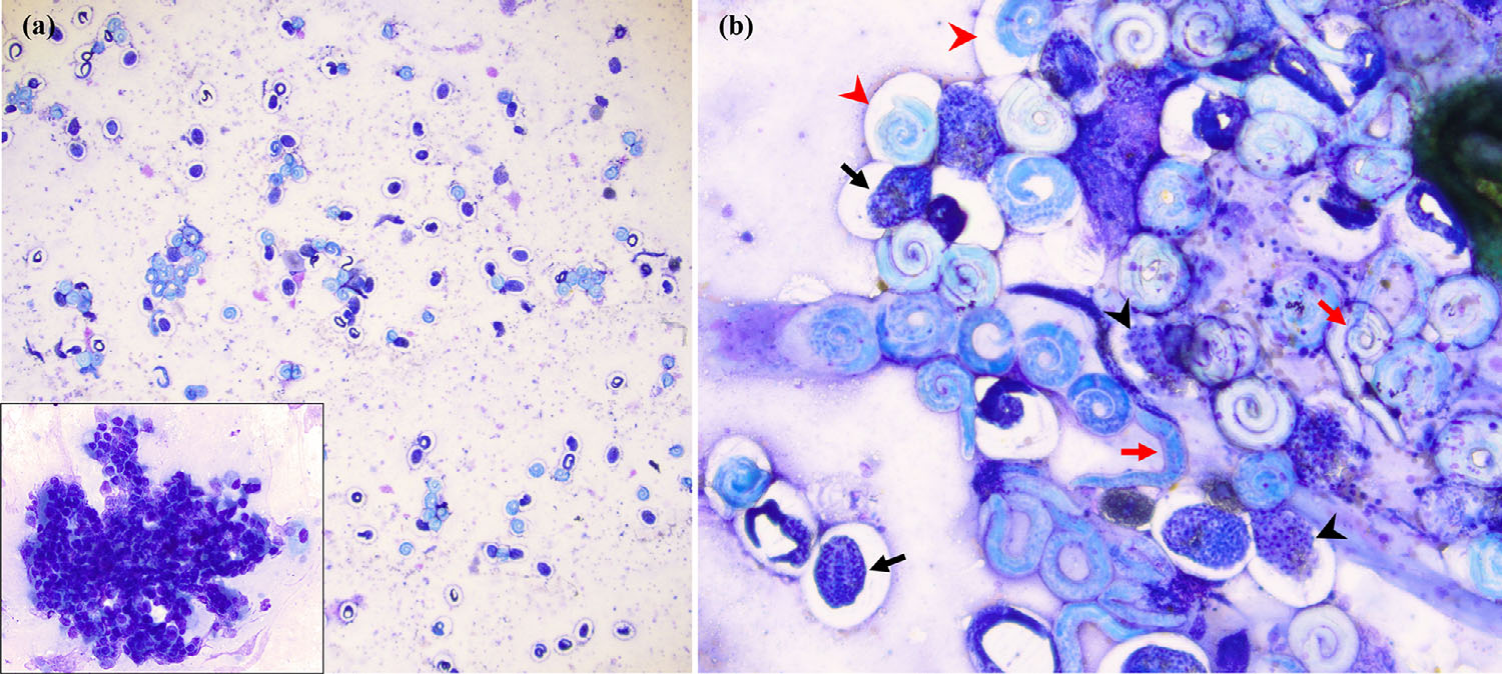
Cytologic findings in the submitted BAL preparations from Case 1; modified Wright stain. (a) Large numbers of coiled larvae and larvated eggs, with moderate numbers of degenerate neutrophils (direct smear, 4x). Insert: variably mature nucleated epithelial cells in clusters interpreted as secondary hyperplasia (direct smear; 50x). (b) Mixed immature forms (see Section 4), including presumed zygote gone through a few cell divisions (black arrow heads), presumed multicelled embryo at the tissue/organ development stage (black arrows), larvated ova (red arrow heads) and free larvae (red arrows) (concentrated preparation, 20x).

**FIGURE 2:**
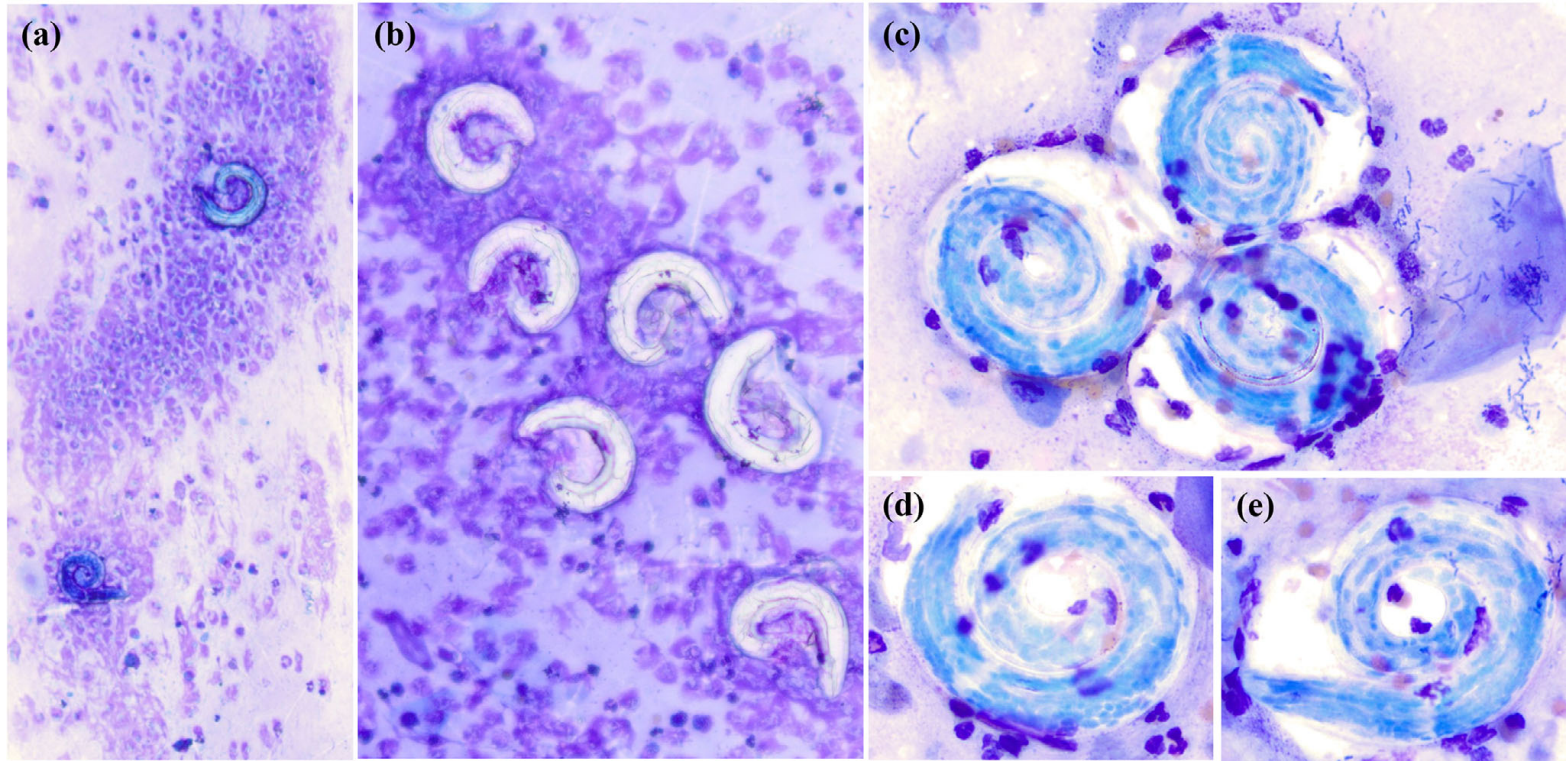
Cytologic findings in the submitted BAL preparations from Case 1; modified Wright stain. (a) Neutrophils concentrated around larvae (direct smear, 10x). (b) Neutrophils concentrated around larvae (direct smear, 20x). (c–e) Neutrophils concentrated on larvae (direct smear, 50x). Panel (c) also shows mixed bacteria strictly extracellular, occasionally on anucleated squamous epithelial cells.

**FIGURE 3:**
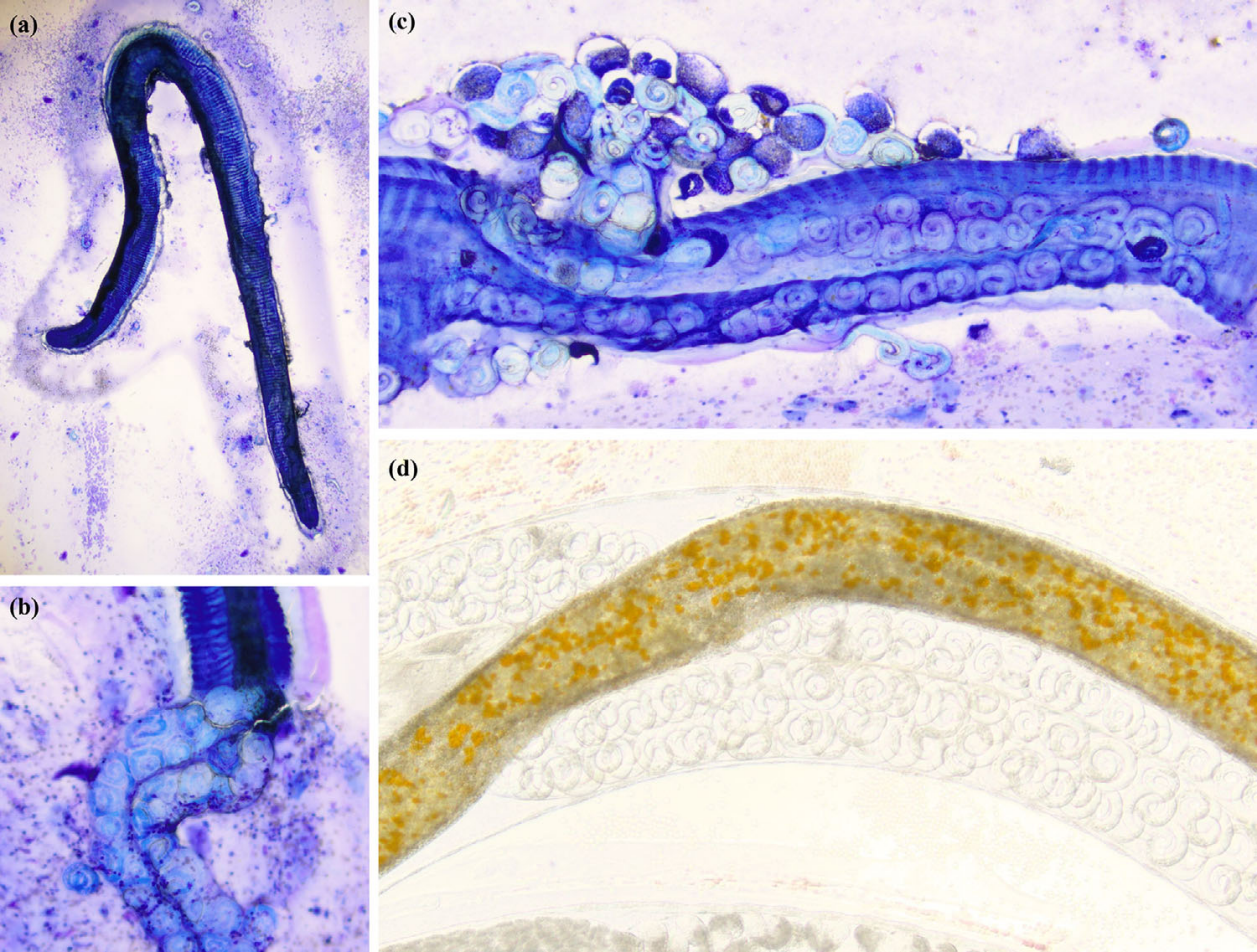
Cytologic findings in the submitted BAL preparations from Case 1. (a) Intact adult female nematode, >1 cm (modified Wright stain, direct smear, 4x). (b) Fragmented adult female nematode, containing numerous immature forms (modified Wright stain, direct smear, 10x). (c) Fragmented adult female nematode, containing numerous immature forms. The juxtaposed spread content reveals a mix of immature forms, similar to observations on the concentrated preparation (Figure 1b) (modified Wright stain, direct smear, 10x). (d) Partial view of an intact female adult loaded with immature forms (unstained, wet preparation, direct smear, 10x).

Cytologic examination was performed on submitted direct smears, dry direct and concentrated preparations from the submitted fluid, and unstained wet preparations from the submitted fluid. Both direct (Figure 1a) and concentrated (Figure 1b) preparations were at least moderately cellular and consisted of large numbers of often coiled nematode larvae (250 µm × 30 µm), along with moderate numbers of markedly degenerate neutrophils, occasional aggregates of macrophages, moderate numbers of clustered epithelial cells (Figure 1a, insert) and large numbers of highly diverse bacteria. There were mixed immature nematode forms (Figure 1b), including larvae and earlier stages. Numerous basophilic granular structures were noted within the larvae. Neutrophils were typically concentrated around/on the larvae (Figure 2). Bacteria were typically grouped on or around large anucleated squamous epithelial cells and were not observed intracellularly (Figure 2c). Adult nematodes were observed (Figure 3), either intact or in fragmented forms, and contained numerous developing parasites. On wet preparations (Figure 3d) of the submitted fluid, an adult worm measuring >1 cm in length and >500 µm in thickness was identified and contained numerous larvae within its reproductive tract. The cytologic interpretation was as follows: severe verminous pneumonia, with neutrophilic and macrophagic inflammation, and reactive epithelial hyperplasia. The inflammation and the hyperplasia were interpreted as secondary to the severe nematode infection, whereas the bacterial presence was attributed to most likely contamination of the sample, based on the diversity and distribution of the bacteria, mostly on anucleated squamous epithelial cells, while never intracellular. Concerning the parasitic aetiology, *Filaroides* spp. was suspected based on pathophysiologic and morphologic considerations. Morphologic examination did not allow for the differentiation between *O. osleri* and *F. hirthi* (grouped together under *Filaroides* spp.). Several stained slides containing numerous larvae were used for subsequent PCR investigation. BAL bacterial culture was also selected and yielded moderate growth of mixed bacterial species.

Following the cytologic evaluation, the patient was started on fenbendazole (Panacur) (50 mg/kg/day PO for 4 weeks) and prednisone (0.8 mg/kg/day PO for 14 days, then tapered until recheck). At this time, the patient also received heartworm preventative (Heartgard 51–100 lbs.) consisting of ivermectin (4.5 µg/kg PO) and pyrantel (8.4 mg/kg PO). Moreover, amoxicillin (18.5 mg/kg PO twice a day for 4 days) and clavulanic acid (4.6 mg/kg PO twice a day for 4 days) (Clavamox) were added after the BAL fluid culture was received, despite cytologic interpretation as incidental. The patient improved moderately, though coughing episodes continued in phases of excitement. Three months later, another course of fenbendazole was initiated due to the patient coughing more frequently. Coproscopy by modified double centrifugal flotation (MDCF) was performed; the Baermann test was added to rule out other potential differentials. No eggs or larvae were detected. The patient continued immunotherapy for environmental allergen hyposensitisation but did not undergo additional testing or treatment.

### 2.2 Genomic DNA Extraction From BAL Smear

Genomic DNA was extracted from stained BAL smears that had numerous lungworm larvae (Silvestre and Humbert 2000; Zhang et al. 2006). Approximately 200 µL of TE (1 mM Tris–0.1 mM EDTA) buffer was placed on the smear, and the cytologic material was gently scraped off into the buffer solution using a sterile pipette tip. This solution containing the scraped cytologic material was collected into a microcentrifuge tube and spun at 100 × *g* for 5 min. The supernatant was removed, and the pellet was retained. Pellets were resuspended in 100 µL of DNA extraction buffer (1 mM Tris, 0.1 mM EDTA, 5 mg/mL proteinase K), followed by overnight incubation at 55°C to digest the parasites and release genomic DNA. Proteinase K was inactivated by incubating the genomic DNA preparation at 95°C for 20 min, making it suitable for use as a template in PCR reactions. A summary of the cases is provided in Table 1.

**TABLE 1:** Summaries of Cases 1 and 2.

	Case 1	Case 2
Animal	2.5‐year‐old spayed female English Bulldog	2‐year‐old castrated male mixed‐breed dog
History	Chronic dermatologic issues attributed to environmental allergy, chronic cough and haemoptysis	Chronic cough for 3 months
Prior medication	Oclacitinib (Apoquel), repeated courses of tapered prednisone	Trimeprazine combined with prednisolone (Temaril‐P), prednisone (12‐day taper) and chlorpheniramine; unsuccessful
Complaint	Haemoptysis for 3 weeks	Chronic cough
Physical examination	Faint, dry, non‐productive cough every few seconds; harsh lung sounds	Initially unremarkable: later, ‘popping’ sounds on inspiration during pulmonary auscultation
Biochemistry	Unremarkable	Unremarkable
CBC	Mild eosinophilia: 1.88 × 10^9^/L [0.06 – 1.23 × 10^9^/L]	Unremarkable
SNAP test	4Dx Plus test: negative	Not performed
Bronchoscopy	Results unavailable at the time of cytology interpretation; trachea hyperaemic, numerous nodules containing nematodes	Multiple round mucosal nodules at the tracheal bifurcation containing white nematode organisms
Ancillary	BAL cytology: severe nematodiasis (*Filaroides* spp.), with neutrophilic and macrophagic inflammation	Tracheal nodule histopathology: marked multifocal chronic lymphohistiocytic bronchitis with intralesional adult nematodes consistent with *O. osleri*
Faecal testing	Not performed	Baermann test: negative
PCR	On scraped material from BAL smears: consistent with *O. osleri* and 100% identical to Case 2	On FFPE tissue sections of the tracheal nodule: consistent with *O. osleri* and 100% identical to Case 1
Treatment	Fenbendazole (Panacur) (50 mg/kg/day PO for 4 weeks) and prednisone (0.8 mg/kg/day PO for 14 days, then tapered until recheck)	Fenbendazole alone (50 mg/kg/day PO for 3 weeks)
Outcome	Moderate improvement; another course of fenbendazole 3 months later due to coughing more frequently	Clinical resolution, no subsequent clinical signs (no recurrence over 1.5 years)
Faecal testing posttreatment	Coproscopy and Baermann: no parasite detected	Not performed

### 2.3 Primer Design and PCR Testing

Several primers (Table 2; Figure 6) were designed using *O. osleri* ribosomal RNA gene sequences available in GenBank (accession numbers: AY295812, MT232878, MN227205). The NCBI (National Center for Biotechnology Information) primer blast tool (www.ncbi.nlm.nih.gov/tools/primerblast) was used to obtain primer pairs that would amplify different regions of the ribosomal RNA gene. Conventional PCR reactions were set up using 5x GoTaqFlexi buffer, 2.5 mM magnesium chloride solution, 0.2 mM PCR nucleotide mix, 1 µM of each upstream and downstream primer and 2 U of GoTaq DNA Polymerase (Promega, Madison). Amplifications were performed in a T100 thermal cycler (Bio‐Rad Laboratories Inc., California). Approximately 5–10 µL of each sample was used as a template for 50 µL PCR reactions. The thermal cycling parameters included an initial denaturation at 95°C for 5 min, followed by 40 cycles of denaturation at 95°C for 30 s, an annealing temperature specific for the primer pairs, and extension at 72°C. After amplification, a single final extension was performed at 72°C. The amplified PCR products from each of the primer pairs were visualised on ethidium bromide‐stained agarose gels.

**TABLE 2:** Primers designed for amplification of *Oslerus osleri* rRNA regions and their sequence similarity to corresponding GenBank entries. Primers were designated as Oos_Fx and Oos_Rx, where ‘Oos’ denotes *O. osleri*, ‘F’ and ‘R’ indicate forward and reverse primers, respectively, and ‘x’ (1–6) represents the primer number.

Primer name	Primer sequence (5ʹ–3ʹ)	Target region (rRNA gene, GenBank ID)	Amplicon size (bp)	Per cent identity between amplicons from BAL and FFPE‐derived gDNA	NCBI BLAST search results	
					Organism	Percent identity
Oos_F1 Oos_R1	F: AGCCATGCATGTCGAGTTCA R: GATAAGTTTCGCGCCTGCTG	18S (AY295812)	378	100	*O. osleri*	100
Oos_F2 Oos_R2	F: AACGGCTACCACATCCAAGG R: ACAATACCCCATCGGATCGC	18S (AY295812)	646	100	*O. osleri*	100
Oos_F3 Oos_R3	F: GGAGCCTGCGGCTTAATTTG R: CGGTTCAAGCCATTGCGATT	18S (AY295812)	576	100	*O. osleri*	100
Oos_F4 Oos_R4	F: GGACGCCCTAAAGCGAAAGC R: CCAGACAAATCGCTCCACCA	18S (AY295812)	383	100	*O. osleri*	100
Oos_F5 Oos_R5	F: CGGGTAAAAGTCGTAACAAGGTA R: AGTTGTCTTCAACAACCCTGAA	Partial 18S, ITS1 and partial 5.8S (KP987220)	924^a^	100	*O. rostratus*	80.65
Oos_F6 Oos_R6	F: CTGGTTCAGGGTTGTTGAAGAC R: GTTTGCTATCGAATTCGCGTCC	Partial 5.8S, ITS2 and partial 28S(MT232878, MN227205)	817	100	*O. osleri*	98.66, 99.65

^a^ New sequence generated during this study.

### 2.4 Amplicon Sequencing Protocol

The individual PCR products were purified using NucleoSpin Gel and PCR Clean‐up kit (Macherey‐Nagel, Germany) and submitted for Sanger sequencing (Eurofins Genomics, Kentucky). The obtained nucleotide sequences were analysed using BLAST (https://blast.ncbi.nlm.nih.gov/Blast.cgi) search services available at NCBI.

### 2.5 Amplicon Sequencing Results

Amplicons of expected sizes were successfully amplified from genomic DNA extracted from BAL smears (Figure 7a,b) using primer pairs listed in Table 2. BLAST analysis of the nucleotide sequences of each amplicon obtained from Sanger sequencing of the PCR products showed 100% identity to 18S and 28S ribosomal RNA gene sequences of *O. osleri* recovered from a coyote from California (Carreno and Nadler 2003) (GenBank accession: AY295812, AY292800). The GenBank currently lacks nucleotide sequence information for *O. osleri* ITS1 and 5.8S regions of the ribosomal RNA gene. In the absence of this information, the nucleotide sequences of ITS 1 and 5.8S generated in this study showed 78.65% and 99.33% identity to corresponding sequences from *Oslerus rostratus*.

## 3 Case 2

### 3.1 Clinical Report

A 2‐year‐old castrated male mixed‐breed dog weighing 16.8 kg was presented to a veterinary speciality hospital for constant wheezing and coughing. The patient was adopted 6 months prior and started developing clinical signs 3 months ago. The dog's history before adoption, including place of origin, was not reported. Thoracic radiographs performed by the primary veterinarian revealed ‘increased airway visibility’ with no signs of pneumonia. The patient was treated with trimeprazine combined with prednisolone (Temaril‐P) for 2 weeks, with no clinical response. A trial of prednisone (tapering protocol over 12 days) along with chlorpheniramine (an antihistamine) was then performed, initially improving the clinical signs, but the cough returned within the following few weeks. Two months later, prednisone was repeated, with no response. It was then advised to seek an internal medicine consultation.

On admission to internal medicine consultation, the patient had no known travel history outside of Colorado and was up to date on vaccines. There was no sneezing or nasal discharge, and the patient had a normal reported appetite and elimination. Physical examination was normal. CBC and serum chemistry were performed and were unremarkable. Thoracic radiographs showed a diffuse bronchial pattern, particularly in the caudal peripheral lung lobes. Cardiovascular and osseous structures were normal. Given the pulmonary radiographic changes with no physical or haematologic/biochemical abnormalities, it was recommended to pursue a tracheobronchoscopy.

**FIGURE 4:**
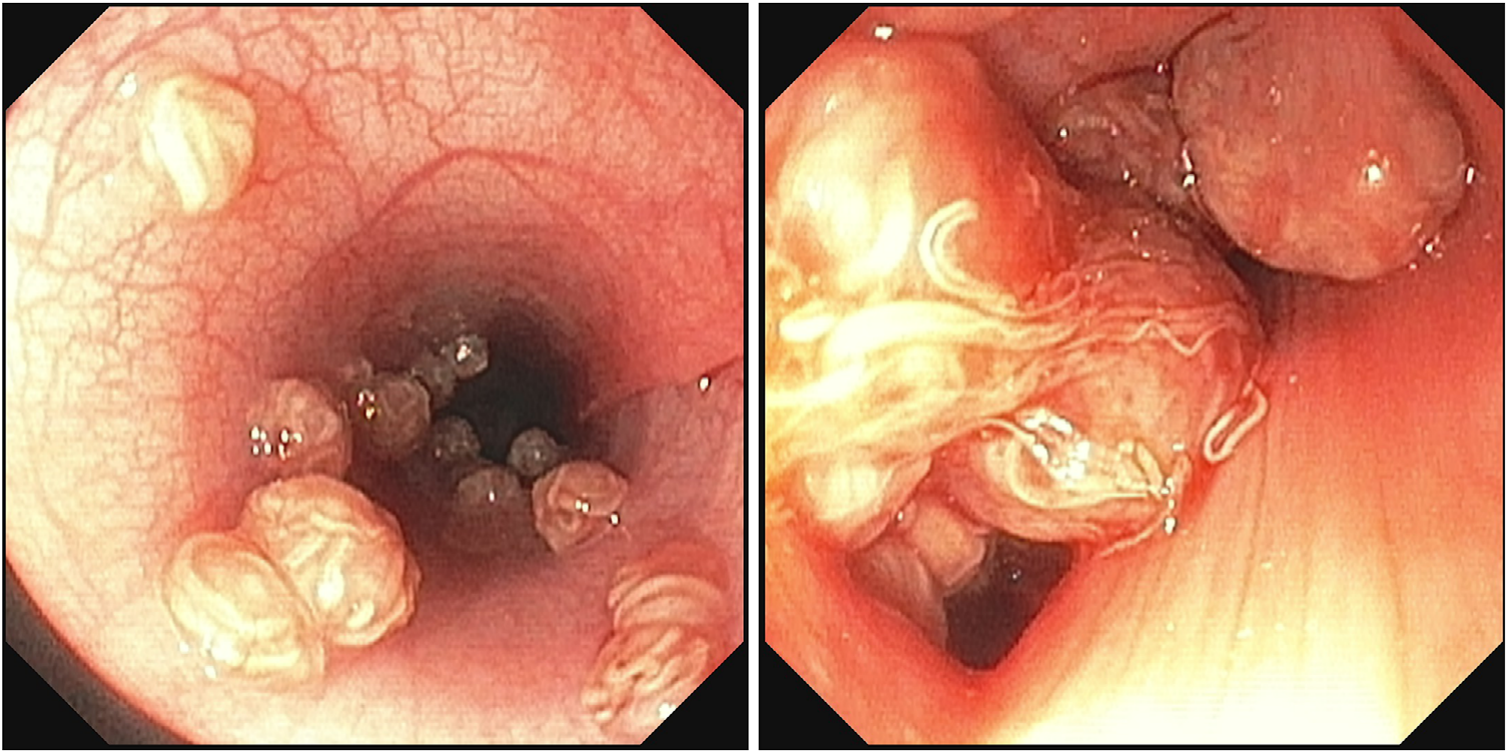
Bronchoscopic images of Case 2, showing multiple tracheobronchial nodules, distended by numerous *Oslerus osleri* nematodes.

The patient was returned 1 week later for the procedure. Physical examination this time revealed ‘popping’ sounds on inspiration during pulmonary auscultation, but no other physical examination abnormalities appeared. The trachea‐bronchoscopy revealed multiple round mucosal nodules at the tracheal bifurcation containing white nematode organisms (Figure 4). The number of nodules increased approaching the carina. A loop retriever was used to debulk one nodule, which was submitted en masse for histopathology. BAL was not performed. A Baermann test was conducted to rule out additional parasites, and no larvae were detected. The patient recovered without complication.

**FIGURE 5:**
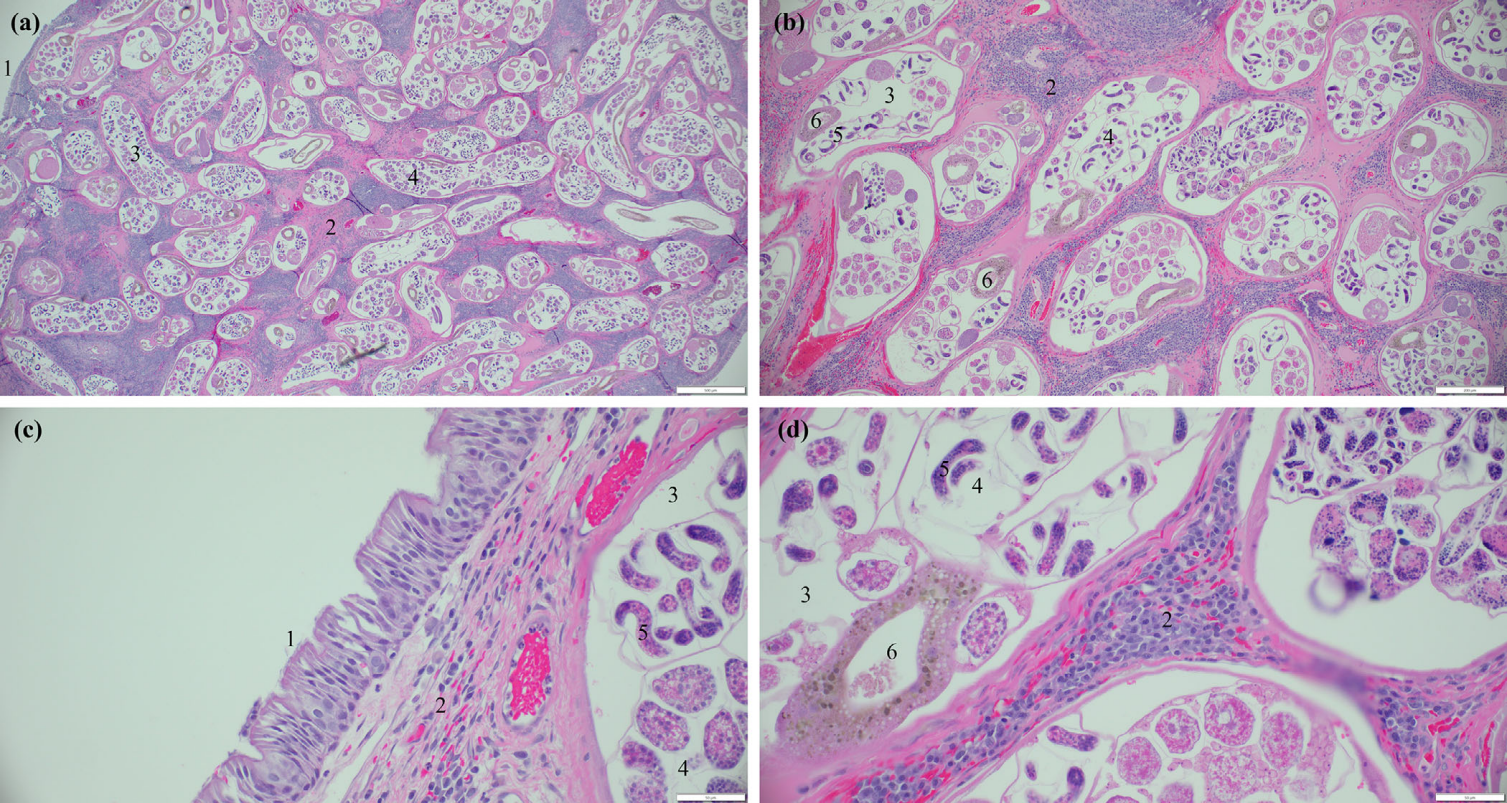
Photomicrographs of histologic sections from the tracheal nodules in Case 2. Haematoxylin and eosin‐stained sections of nodules from bronchoscopy. (a, b) Multiple longitudinal and cross‐sections of adult nematodes within the inflamed parenchyma. (c) The nodule is lined by ciliated respiratory epithelium. (d) Increased magnification demonstrating lymphohistiocytic inflammation. Legends: (1) ciliated respiratory epithelium; (2) inflamed parenchyma; (3) adult nematode; (4) reproductive tract of the adult nematode; (5) immature forms within the reproductive tract; and (6) intestine of the adult nematode.

On histopathologic examination (Figure 5), there was abundant histiocytic and lymphoplasmacytic inflammation, along with rare eosinophils surrounding myriad cross and longitudinal sections of adult nematodes, up to 0.4 mm in diameter, in the subepithelial area. The nematodes had a thin, 4‐µm cuticle, coelomyarian musculature, a pseudocoelom with an intestinal tract lined by a single layer of brown‐pigmented epithelium and a reproductive tract that contained innumerable mixed immature forms, including larvae. The submitted nodule was lined by respiratory epithelium. The histopathologic interpretation was as follows: lymphohistiocytic bronchitis, multifocal, chronic, marked, with intralesional adult nematodes consistent with *O. osleri*. Inflammation and hyperplasia were interpreted as occurring secondary to the massive parasitic nematode infection. The interpretation of *O. osleri* was also based on the gross morphology at bronchoscopy and the location of the nodular lesions. Several formalin‐fixed paraffin‐embedded (FFPE) tissue sections were subsequently used for PCR testing for *O. osleri*.

The patient was discharged with fenbendazole treatment (50 mg/kg PO once daily for 3 weeks). It was advised to monitor the patient for signs of anaphylaxis after starting the antiparasitic treatment. Two weeks after discharge, the patient was already markedly improved. At the end of the 3 weeks of treatment, the patient was reported to have no clinical signs. No subsequent related clinical signs were reported in the 1.5 years that followed. A summary of the case is provided in Table 1.

### 3.2 Genomic DNA Extraction From FFPE Sections

Genomic DNA was extracted from FFPE tissue sections containing parasite stages (Silvestre and Humbert 2000; Zhang et al. 2006). Three 5‐µm FFPE tissue sections placed in a microcentrifuge tube were processed by performing deparaffinisation, followed by thorough washing. The resulting tissue pellet was dried for 30 min at room temperature. Similar to pellets from the BAL sample, pellets from the FFPE tissue section were resuspended in 100 µL of DNA extraction buffer (1 mM Tris, 0.1 mM EDTA, 5 mg/mL proteinase K), followed by overnight incubation at 55°C to digest the parasites and release genomic DNA. Proteinase K was inactivated by incubating the genomic DNA preparation at 95°C for 20 min, making it suitable for use as a template in PCR reactions.

### 3.3 Primer Design and PCR Testing

**FIGURE 6:**
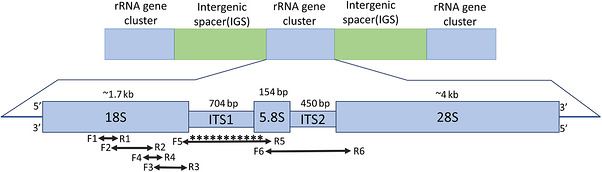
General schematic of the ribosomal RNA gene. The bold double‐headed arrows indicate the approximate positions of primers on the rRNA gene of *Oslerus osleri*. The asterisks represent newly generated sequence information.

Primers and PCR conditions used for BAL samples (Tables 2; Figure 6) were applied unchanged to FFPE tissue sections.

### 3.4 Amplicon Sequencing Protocol

The exact same amplicon sequencing protocol was used in both cases.

### 3.5 Amplicon Sequencing Results

**FIGURE 7:**
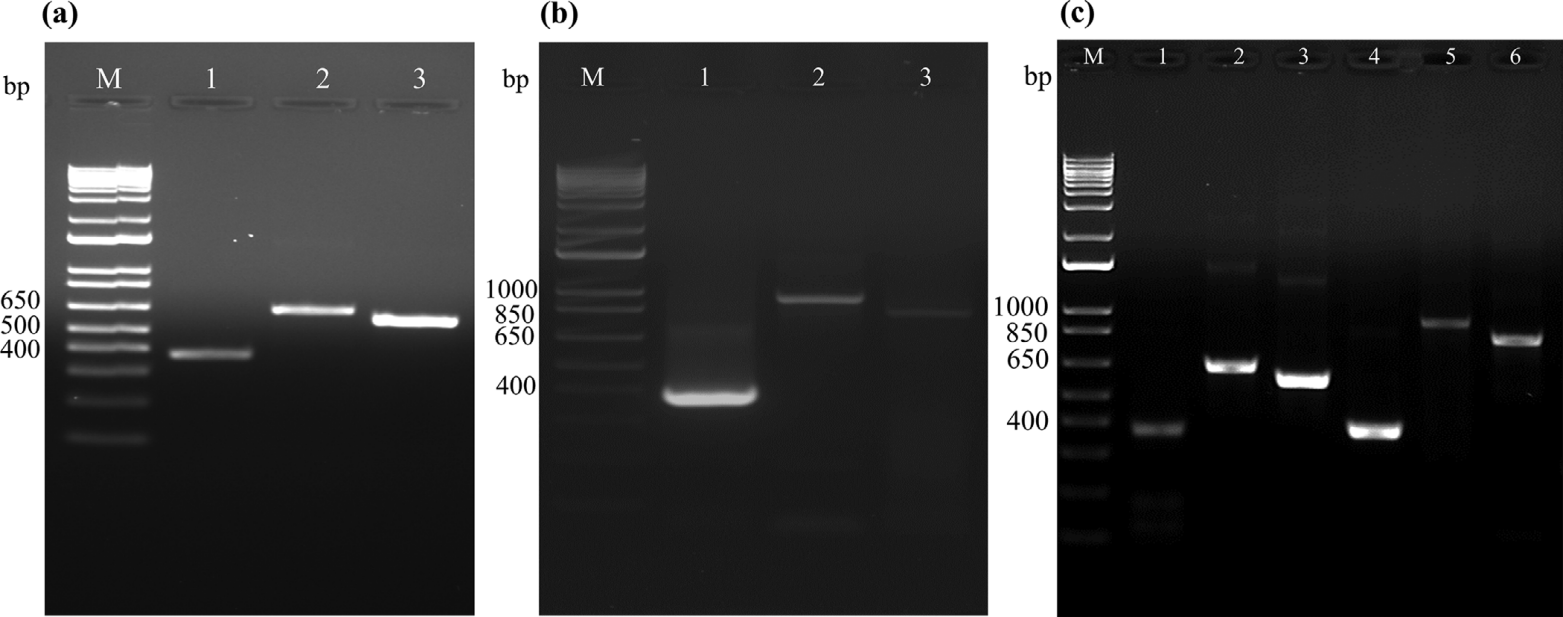
Agarose gel electrophoresis of PCR‐amplified *Oslerus osleri* rRNA gene subunit products obtained from BAL (a, b) and from FFPE tissue sections containing the parasite (c), using different primer pairs. The size of the PCR product amplified using a specific primer pair is provided in parentheses. (a) Lane M: 1‐Kb DNA ladder; Lane 1: F1–R1 (378 bp); Lane 2: F2–R2 (646 bp); Lane 3: F3–R3 (576 bp). (b) Lane M: 1‐Kb DNA ladder; Lane 1: F4–R4 (383 bp); Lane 2: F5–R5 (924 bp); Lane 3: F6–R6 (817 bp). (c) Lane M: 1‐Kb DNA ladder; Lane 1: F1–R1 (378 bp); Lane 2: F2–R2 (646 bp); Lane 3: F3–R3 (576 bp); Lane 4: F4–R4 (383 bp); Lane 5: F5–R5 (924 bp); Lane 6: F6–R6 (817 bp).

Amplicons of expected sizes were successfully amplified from genomic DNA extracted from FFPE tissue sections (Figure 7c). BLAST analysis of the nucleotide sequences of each amplicon obtained from Sanger sequencing of the PCR products showed 100% identity between both cases. The overall nucleotide analysis indicated that the nematodes present in the BAL and FFPE specimens were *O. osleri*.

## 4 Discussion

The primary aim of this study was to demonstrate the feasibility of PCR‐based molecular identification of *O. osleri* obtained from typically available diagnostic materials, including stained BAL cytology smears and FFPE tissue sections. A recent study validated a multiplex qPCR assay for several canine lungworms (Massetti et al. 2024), including *Angiostrongylus vasorum*, *Crenosoma vulpis*, *Eucoleus aerophilus* and *Eucoleus boehmi*; however, species within the genus *Filaroides* (such as *O. osleri* and *F. hirthi*) were not included. We performed PCR testing on genomic DNA extracted from stained smears/cytology slides and FFPE tissue sections, using custom‐synthesised primers. First, the size and sequence of the amplicons obtained from the BAL sample and FFPE tissue sections from Case 1 and Case 2, respectively, had 100% similarity with each other, supporting that the nematodes from both cases were the same species. The diagnosis of *O. osleri* was certain in Case 2 based on bronchoscopic and histopathologic findings, and there was 100% similarity in sequences between the worms of the two cases, so that *O. osleri* was also confirmed in Case 1.

Second, both cases matched at 100% nucleotide identity to the 18S and 28S sequences of *O. osleri* available in GenBank, confirming our appropriate species identification. Yet, we acknowledge that the specificity of these primers is currently unknown, as they have not yet been tested on other metastrongylid larvae. Investigating the latter is highly warranted, especially by performing the same PCR testing on confirmed cases of *F. hirthi* infections, which may be challenging to obtain given the lower prevalence of this infection. In our study, using the primer combination Oos_F5 and Oos_R5 located on the 18S and 5.8S regions of the rRNA gene, respectively, we obtained the sequence of ITS1 for *O. osleri*. This is a new contribution, as the sequence of this region was previously unavailable. The ITS regions of the rRNA gene are variable between species of the same genus, so it is unsurprising that it shows only 78.65% nucleotide identity to ITS 1 of *O. rostratus*. While a recommendation for a primer pair cannot be provided without performing sensitivity and specificity studies, the primer pairs designed for this study will be useful for molecular identification of *O. osleri*.

The development of new tools for molecular characterisation of *O. osleri* is especially relevant. Despite epidemiologic and clinical factors, the diagnosis of *Oslerus* sp. infection is commonly delayed for multiple reasons, such as the low prevalence of the disease, the limited usefulness of bloodwork and faecal work, or the absence of visual endoscopic input, among others. Similar to another published report of two canine cases of *O. osleri* infection (Yao et al. 2011), the two cases that we are reporting occurred in 1‐and 2‐year‐old dogs, suggesting that *O. osleri* should be considered a differential diagnosis for chronic respiratory diseases in dogs of this age cohort. The clinical manifestations tend to be worse in dogs between 6 and 12 months of age (Barr and Bowman 2011; Taylor et al. 2016). Many infections progress slowly, although some cases may present with an uncommon, more acute onset of dyspnoea with no history of chronic cough (Reagan and Aronsohn 2012). A chronic cough of 6 months or longer remains the most typical clinical sign, as seen in Case 2. The clinical presentation may be confounded with other conditions such as allergies or kennel cough (e.g., Case 1, and some of the previously published studies [Yao et al. 2011]). Thus, in cases of chronic, non‐resolving respiratory signs, diagnostic investigation may benefit from an endotracheal wash or a BAL, with molecular testing providing a valuable means of reaching a diagnosis quickly, particularly when endoscopy is not performed or yields inconclusive findings.

CBC and serum chemistry are useful as an early investigation step for many diseases, although abnormal findings usually remain minimal in cases of *O. osleri* infection. Haematologic changes are described as mild and non‐specific, and peripheral eosinophilia may or may not be detected (Conboy and Jane 2021). In our reports, Case 1 only had mild peripheral eosinophilia at 1.9 × 10^9^/L (RI: 0.1–1.2 × 10^9^/L), and Case 2 had no haematologic abnormalities. Another parasitic differential for chronic cough is *Dirofilaria immitis* (heartworm): a SNAP 4Dx Plus (IDEXX) screening test detecting heartworm antigen (Kotwa et al. 2020) was performed in Case 1 and yielded no antigen detection.

For several reasons, faecal tests for *O. osleri* in infected dogs often result in false‐negative results. MDCF was performed in Case 1, and no parasites were detected. Coproscopic procedures performed in other published cases are also often reported as negative (Yao et al. 2011). Faecal detection of *O. osleri* infection is challenging and highly dependent on the stage of the infection and the faecal test employed. Clinical signs can also occur during the prepatent period (i.e., before the larvae are shed in the faeces) (Verocai et al. 2013; Yao et al. 2011). While the Baermann technique is typically recommended for the detection of larvae, false‐negative results are common for *Filaroides* canine lungworm infections, as the technique relies on active (motile) larvae, whereas larvae from these nematodes are relatively inactive. The larvae of *F. hirthi* and *O. osleri* may best be recovered by performing a zinc sulphate centrifugal faecal flotation test (King 2004). Even with the use of an appropriate testing modality, false negatives can still occur due to intermittent shedding of the larvae. Thus, examination of both BAL fluid and faecal samples may be beneficial in increasing the overall sensitivity of the diagnostic approach.

When identifying parasite larvae by BAL cytologic examination in a dog, several worms may be considered, and differentials may be sorted to narrow down the few ones to consider. Differential diagnoses were successively excluded by integrating parasite taxonomy, host specificity, predilection sites and morphological characteristics. *Paragonimus kellicotti* was excluded because of the class (trematode), *Dirofilaria immitis* because of its intravascular rather than intra‐airway location, *Aelurostrongylus abstrusus* because of the canine host, *Eucoleus aerophilus* (syn. *Capillaria aerophila*) because it produces ova rather than larvae, *A. vasorum* because of its cardiac location and the recovery of several adult worms in the BAL fluid and *Cr. vulpis* because the larvae lacked the characteristic tapered tail morphology of this species. Accordingly, the differential diagnosis was narrowed to *Filaroides* lungworms, including *O. osleri* and *F. hirthi*, prompting the use of molecular characterisation as a diagnostic tool.

Some sources of the literature report that the morphological differentiation of the L1 larval stage of *O. osleri* and *F. hirthi* is feasible based on the presence of a subtle dorsal indentation closer to the tip of the larval tail (McGarry and Morgan 2009) or based on other complex, subtle morphological criteria (Taylor et al. 2016). However, other sources report that morphological differentiation among *Filaroides* L1 larvae is challenging (Torgerson et al. 1997) or even that ‘the L1s of *F. hirthi*, *F. milksi*, and *O. osleri* are morphologically identical and cannot be differentiated from each other’ (Cervone et al. 2018). This may depend on the type of preparations; in routine cytologic examination, this difference may not always be discernible, so that they are sometimes considered indistinguishable indeed. Larvae of other metastrongylids infecting dogs, such as *A. vasorum* and *Cr. vulpis*, are more easily morphologically distinguishable (Starbird et al. 2024).

Distinguishing *O. osleri* from *F. hirthi* is clinically relevant because their biology and clinical implications differ. Some sources consider *O. osleri* as the pathogenic species, whereas other *Filaroides* are considered subclinical (Taylor et al. 2016). Other sources emphasise that despite the fact that infection by *F. hirthi* is often subclinical in immunocompetent dogs, severe clinical disease to fatal outcome may also happen in rare cases, such as cases of massive parasitic infection and/or especially those with immunosuppressive conditions, such as medication by corticosteroids or other immunomodulatory therapies, or additional concomitant chronic disease (Cervone et al. 2018). Regardless, the medical consensus remains to determine the species of the infective nematode. To distinguish between *O. osleri* and *F. hirthi* larvae, PCR testing appears especially relevant. Admittedly, the location of lesions can be pathognomonic for differentiating *O. osleri* (tracheal nodules) from *F. hirthi* (lung parenchyma); however, a PCR test would be highly convenient in a clinical context when larvae are found in the faeces or in a respiratory wash sample, as imaging and/or endoscopy with histopathology may not always be available and may be more invasive and expensive. Beyond individual case management, molecular confirmation improves diagnostic confidence, strengthens case documentation and supports surveillance efforts, which is essential for understanding prevalence and geographic distribution.

Another challenge consists of sorting the various immature forms identified on the BAL preparation (Case 1). *Oslerus osleri* is typically described as ovoviviparous, wherein the females give rise to thin‐walled larvated eggs hatching within the host (Bowman 2020), as opposed to oviparous worms that lay eggs. The presence of immature forms in the BAL fluid (Figure 1) is presumably derived from ruptured adults (Figure 3). Immature forms evolve over a continuum, and larvae can take on different appearances depending on the state of development and condition, as they relate to the diagnostic technique (Bowman 2020; Zajac et al. 2021). Hence, it is challenging to accurately identify and label the ‘morula’ versus the ‘vermiform‐embryo’ stage, as these forms change over time with each cell division (Bowman 2020). The term ‘morula’ is attributed to a ‘zygote that has gone through a few cell divisions’, and the term ‘vermiform‐embryo’ stage is attributed to a ‘multicellular embryo at the tissue/organ development stage’ (Bowman 2020; Kahn and Line 2010; Zajac et al. 2021); hence, our proposed labelling in Figure 1. The morula stage is often referred to as ‘morulated egg’ (Zajac et al. 2021). On the other hand, it is more controversial to refer to the vermiform‐embryo stage as an ‘embryonated egg’ (Starbird et al. 2024). Published descriptions of early immature stages of *O. osleri* on cytologic preparations appear to be scarce.

The inflammatory patterns were relatively different between the BAL fluid cytology (Case 1) and the tracheobronchial nodule histopathology (Case 2). The first one showed a predominant neutrophilic inflammation with no significant lymphoid or eosinophilic components, whereas the second one showed an interstitial predominantly lymphoplasmacytic and histiocytic inflammation with rare eosinophils and no neutrophils. Several considerations may explain these differences. First, we cannot completely rule out concurrent bacterial infection in Case 1, though this appears rather unlikely given the mixed nature of the bacteria, their distribution mostly on squamous epithelial cells and especially the absence of phagocytised bacteria; this combination rather supports sample contamination from the pharyngeal flora during sampling. Second, the inflammatory patterns in alveoli (Case 1) and in the interstitium (Case 2) are not expected to be similar, as the BAL does not recover interstitial inflammatory cells. Third, there could be an acute on‐chronic inflammation component in Case 1 that is not present in Case 2: the trachea was described as much more hyperaemic in Case 1. Most importantly, fourth, only Case 1 received chronic corticotherapy, which is anti‐eosinophilic and anti‐lymphocytic, leaving mostly a neutrophilic inflammatory pattern.

Despite lack of consensus regarding the treatment of *O. osleri* infection in dogs (Conboy and Jane 2021), the current medical practice involves the use of fenbendazole (50 mg/kg/day PO for 14 days, with potentially a second course of 14 days if needed), or high‐dose ivermectin (0.4 mg/kg SC, repeated every 3 weeks for four treatments) if the infection is heavy and fenbendazole alone may not be sufficient (Conboy and Jane 2021; Levitan et al. 1996; Rafailov et al. 2021). While heartworm preventatives containing ivermectin, such as Heartgard, are considered safe even for dogs with the multidrug resistance gene mutation MDR1, the higher dose of ivermectin needed for the treatment of *O. osleri* infection may not be safe for MDR1 dogs. In this study, Case 1 and Case 2 were treated similarly with an adequate dose of fenbendazole (50 mg/kg/day PO for 4 and 3 weeks, respectively). In the two‐case report from Yao et al., both cases were also treated with fenbendazole 50 mg/kg/day (one for 14 days and one for 26 days [Yao et al. 2011]). Despite excellent clinical remission in their two cases, an endoscopy in their second case, 20 months after treatment, showed a few small remnant nodules at the tracheal bifurcation, emphasising the difference between clinical remission (achieved) and parasite clearance. It is especially relevant to emphasise the specific high dosage of fenbendazole and ivermectin needed for the treatment of *O. osleri*. Indeed, using fenbendazole for the treatment of *O. osleri* is recommended at 50 mg/kg/day for 14 days (Cervone et al. 2018; Husnik et al. 2016; Rafailov et al. 2021), whereas GI helminth infections are often addressed with significantly lower dosing regimens. Failure of treatment could result from the inappropriate dosing. Similarly, ivermectin for the treatment of *O. osleri* is recommended at 0.4 mg/kg SC every 3 weeks for 4 weeks (Rafailov et al. 2021), whereas Heartgard (ivermectin/pyrantel) for dogs 51–100 lbs. (23–45 kg) contains only 0.272 mg of ivermectin and is taken PO once, which is inadequately low for the treatment of *O. osleri*. Case 1 received Heartgard at the time of treatment for heartworm prevention, not for lungworm treatment. Moreover, Case 1 initially received corticosteroids for months due to the allergic skin condition, as an allergy was initially suspected to also contribute to the chronic respiratory clinical signs. Chronic corticosteroid medication could potentially have facilitated lungworm development by immunosuppression. Finally, *O. osleri*’s closest morphological cytologic differential is *F. hirthi*, and accurate identification of the species is particularly challenging when endoscopy is not available (or endoscopic findings are not available to the pathologist). Correctly identifying the species at the diagnostic step is relevant, as clearance of the parasite may be harder to achieve in *F. hirthi*, and the prognosis may become worse in specific cases. Hence, differences in the therapeutic strategies are reported in the literature. For *F. hirthi*, the current recommendation is to combine fenbendazole (50 mg/kg/day PO for 14 days) with ivermectin (0.4 mg/kg SQ every 2 weeks for three treatments) (Cervone et al. 2018).

To conclude, we report the cases of two young dogs (1–2 years old) with *O. osleri* infection, presenting with either challenging (Case 1) or straightforward clinical signs (Case 2), with unremarkable to minimal bloodwork changes, and ultimately diagnosed by BAL fluid cytological analysis or histopathologic analysis, respectively. High‐quality samples and pictures are presented, illustrating how challenging the morphological recognition of *O. osleri* versus *F. hirthi* can be in practice. Multiple primer pairs were designed for *O. osleri*, yielding specific amplicons in terms of both sizes and sequences. Assessing the specificity of the primers is warranted for further studies. The questionable utility of the faecal Baermann test and the successful clinical treatment/remission by a high dose of fenbendazole are typical features of *O. osleri* infection management in dogs and are once again illustrated in our report.

## Author Contributions

Jeremie Korchia, Sriveny Dangoudoubiyam, Vishnu Manikantan, and Ashley K McGrew contributed substantially to the conception and design of the study. Clinical and cytologic data reporting for Case 1 was performed by Christina M Jeffries and Jeremie Korchia. Clinical data acquisition for Case 2 was performed by Kelly McCord. Histopathological data reporting for Case 2 was performed by Rebecca L Makii. Molecular analysis on both cases was performed by Sriveny Dangoudoubiyam and Vishnu Manikantan. The initial draft of the manuscript was prepared by Jeremie Korchia, with the contribution of Christina M Jeffries for Case 1, Rebecca L Makii for Case 2, and Sriveny Dangoudoubiyam and Vishnu Manikantan for molecular analysis on both cases. Critical revision for important intellectual content was conducted by Ashley K McGrew, Sriveny Dangoudoubiyam, and Vishnu Manikantan. All authors have reviewed, edited, and approved the final version of the manuscript for publication.

## Funding

The authors have nothing to report.

## Conflicts of Interest

The authors declare no conflicts of interest.

## Data Availability

The data that support the findings of this study are available from the corresponding author upon reasonable request.
